# A study on the incidence, microbiological analysis and investigations on the source of infection of postoperative infectious endophthalmitis in a tertiary care ophthalmic hospital: An 8-year study

**DOI:** 10.4103/0301-4738.64132

**Published:** 2010

**Authors:** Malathi Jambulingam, Suresh Kumar Parameswaran, Sagar Lysa, Margarita Selvaraj, Hajib N Madhavan

**Affiliations:** L & T Microbiology Research Center, Tamil Nadu, India; 1 Department of Nursing, Vision Research Foundation, Sankara Nethralaya, 18, College Road, Chennai - 600 006, Tamil Nadu, India

**Keywords:** Incidence, postoperative infectious endophthalmitis, source of infection

## Abstract

**Background:**

The objective of the study was the determination of the incidence of culture-proven postoperative endophthalmitis and probable sources of infection.

**Materials and Methods:**

It was a prospective study on the microbiology, incidence and probable sources of infection in patients with postoperative infectious endophthalmitis carried out in a tertiary care eye hospital. Consecutive patients diagnosed with postoperative infectious endophthalmitis during the years 2000-2007 were investigated for the causative infective agent and possible sources of infection. The surgical data and microbiological data including the investigations performed to trace the source were recorded in a specific formatted form and were gathered and compiled for analysis.

**Results:**

Data of analysis showed that 98 (0.042%) out of 2,31,259 patients who underwent intra-ocular surgery developed infectious endophthalmitis. Among these, 70 (0.053%) occurred after cataract, 10 (0.5%) after penetrating keratoplasty (PK) and 18 (0.018%) following other types of intra-ocular surgeries. The predominant infectious agents isolated were bacteria (89.7%), with equal proportions of Gram-positive and Gram-negative bacteria. Polymicrobial infection was noted in four and fungi in seven patients. Occurrence of postoperative endophthalmitis was sporadic and not related to any specific part of period in a year. Sources of infection were donor corneal rim in six post-PK patients and phaco probe in one who had postphacoemulsification endophthalmitis

**Conclusions:**

Overall incidence of postoperative endophthalmitis over an 8-year period was quite low. The sources of infection could be established in six post-PK endophthalmitis patients and in a postcataract surgery.

Postoperative infectious endophthalmitis is a devastating sight-threatening postsurgical ocular complication. Apart from identification of the causative infectious agent, investigations are necessary not only to determine the incidence of this condition but also to identify the sources of such infections, and these are necessary to understand its epidemiology in the given ophthalmic hospital to develop a plan for the prevention of its occurrence. Often, the identity of the infective agent can be presumed with reasonable accuracy depending on the clinical presentation such as acute, delayed and chronic endophthalmitis, depending on the time of onset of symptoms, may be grouped according to the onset of symptoms as acute, which occurs within 6 weeks of surgery, delayed if it is more than 6 weeks and less than 1 year of time and chronic if it is beyond 1 year.[1,2]

Nearly 70% of postoperative infectious endophthalmitis follow cataract surgery.[3] Varying incidence of postoperative infectious endophthalmitis have been reported from different parts of the world, but data of value from India are sparsely available in the literature. There are two reports from south India: one on its incidence following cataract surgeries[4] and another with a description of the relationship between the visual outcome and clinical presentations of postcataract endophthalmitis.[5]

The aim of the present study was to find the incidence of culture-proven postoperative endophthalmitis during an 8-year period and the probable source of infection carried out prospectively at a high volume tertiary care ophthalmic hospital, Chennai, India.

## Materials and Methods

Consecutive patients with complaints of decrease in vision, presenting with hypopyon, vitreous opacification and pain following surgery reporting with a duly filled-in specifically designed form were included in this 8-year study. The information on the patient's general health, preoperative procedures, surgical procedures, phaco probe used, viscoelastics used and surgical complications if any were recorded. For the purposes of this study, postoperative infectious endophthalmitis was considered as established only in patients from whom an infectious agent was isolated from their intra-ocular specimens.[3]

Following standard operating procedures (SOP) were followed at the operation theater room (OT). The theatres were monitored for bacterial load by the settle plate method and slit air sampling methods.[6] The OT atmosphere was disinfected by fogging at the end of the week with 0.25% solution of combination of potassium peroxomonosulfate 50%, sulfamic acid 5% and sodium alkyl benzene sulfonate 15% apart from wet swabbing of the surfaces of walls, floors every day with 1% solution of the same. This combination of disinfectants was decided for use after verification of its microbicidal efficacy.[7] Air filters in air handling units (AHU) were cleaned and fogged with the same bactericidal solution every week. Lint-free fabric for patients and medical-grade disposable tray wrappers to prevent particulate materials floating into the OT atmosphere were used. The immediate floor area 3–4 feet around the OT table was swabbed with the same disinfectant bactericidal solution before every surgery. The potency of the bactericidal solutions was microbiologically tested by the methodology described by Mattila *et al*.[7] To reduce unnecessary entry of persons in the OT and to prevent exposure of surgical supplies to the outside atmosphere, they were passed through closable hatch windows.

For sterilization, pre-vacuum-based autoclaves ensuring complete removal of air pockets in the sterilization chambers were used in the OT and their functioning were monitored daily using both biological *Geobacillus stearothermophilus* (Raven Biological Laboratories, Omaha, USA) and chemical indicators (Signolac, Johnson and Johnson, Thane, India). To ensure steam penetration into the articles placed in the trays, steam integrators were placed in them. All heat-labile tubings were sterilized by ethylene oxide gas and the functioning was monitored daily with *Bacillus atrophaeus* (Raven Bioloical Laboratories). Surgical instruments were processed for cleaning in ultrasonic cleaners using enzymatic solutions and were scanned under magnoscopic examination for detection of any debris sticking on to them. After each surgery, phaco probes were flushed with distilled water in automated rinsing systems and remnants inside phaco tubing were flushed out using high-pressured guns. Phaco probes underwent enzymatic cleaning everyday with further treatment with iso-propyl alcohol once a month. Water that had undergone reverse osmosis was used in the OT with frequent chemical quality checks being conducted.

Apart from the standard practice of cleanliness of the body surface, such as bathing and washing of the face, patients were instructed to instil sulphacetamide 10% eye drops four times a day for 3 days before the planned surgery to reduce the bacterial load of conjunctival flora. On the day of surgery, after skin test verification for hypersensitivity, the patient had intramuscular injection of ampicillin–sulbactum (consists 0.5 g ampicillin and 0.25 g sulbactum) approximately 90–110 min before surgery. This procedure was introduced as a SOP after an earlier research work that demonstrated the presence of high concentrations of these drugs in the anterior chamber in about 90–120 min after an intramuscular injection of the same.[8] A drop of 5% povidone iodine instilled in the conjunctival sac and skin of eyelids and that side of the face was prepared with 10% povidone iodine solution. Lid margins were scrubbed using cotton-tipped applicators dipped in 10% povidone iodine. Five percent povidone iodine was also used to flush the conjunctival cul-d-sac at the conclusion of surgery.

As a standard routine practice of the hospital, the donor corneal scleral rims of all the donor eyeballs used for penetrating keratoplasty (PK) surgeries were cultured as early as possible by placing it in brain heart infusion broth (BHIB) and subcultured when the medium turned turbid for isolation followed by identification and antibiogram performance. The culture report was recorded in the form specifically designed for future analysis.

Ninety eight patients who reported back to the hospital with clinical symptoms and signs of postoperative inflammations mentioned above (under heading "patients") were subjected to diagnostic microbiological investigations to identify the causative agents. The other investigations carried out to trace the source of infection included cytotoxicity test performed with batches of viscoelastics used on patients (only in acute onset), culturing the phaco probe in all phaco surgeries.

The diagnostic aqueous humor and/or vitreous fluid specimen samples were collected from all 98 patients and processed for isolation of the causative infectious agent as described earlier.[9] Isolation of facultative aerobic bacteria was carried out by inoculating onto blood agar (BA), chocolate agar (CA), MacConkey agar, BHIB and anerobic bacteria by inoculating onto Brucella blood agar (BBA) and thioglycolate broth and fungi onto Sabourad's dextrose agar. BA and Mac Conkey agar were incubated at 37°C, CA in 10% CO_2_ atmosphere at 37°C and BBA in the compact anerobic work station. Isolation of similar bacteria/fungi in more than one media was considered positive.

Bacteria and fungi isolated in culture were further identified using conventional microbiological methods.[10] The results of the microbiological investigations were recorded in the specifically designed form.

The following investigations were undertaken to identify the likely source of infection. Cytotoxicity test of the viscoelastic solution belonging to the batch and lot number used for the patient during the surgery was performed on a HeLa cell line as described by us earlier[11] to determine whether the viscoelastic solution could be the cause of inflammation. Cultures of washings from both irrigation and aspiration ports of phacoemulsification probes (record on the identity of each probe used for every patient was maintained in OTs) used for cataract surgeries in these patients were performed as described by us earlier.[12‐14] In case of PK, correlation with the microorganism isolated from donor corneal rim rims of the donor eye was performed.

## Results

A total of 2,31,259 surgeries were performed during this period. The number and types of ophthalmic surgeries during this period were as follows: 1,31,904 were cataract extraction (CE) surgeries, 1,949 PK and the remaining 97,406 were vitreoretinal and other types of intra-ocular surgeries.

Data recorded in the specifically designed form were analyzed at the end of 2007 and found 98 (0.042%) culture-proven postoperative endophthalmitis, 90 (91.8 %) had acute-onset and eight (8.16%) others had delayed-onset postoperative endophthalmitis. Among 1,31,904 cataract surgeries, 70 (0.053%) had postoperative infectious endophthalmitis and the types of cataract surgery performed on these patients were phacoemulsification (PE) in 45, extracapsular cataract extractions (ECCE) in 20 and small incision cataract surgeries in five, all of them with IOL implantation. Ten (0.5%) out of 1949 patients who underwent PK surgeries developed postoperative endophthalmitis. Eighteen (0.018%) of 97,406 other types of intra-ocular surgeries including vitreoretinal surgeries developed infectious endophthalmitis [Tables 1 and 2]. For a better understanding, 18 other postinfectious endophthalmitis were grouped into five categories based on the surgical procedures performed. Group I–vitrectomy with or without other procedures, namely epiretinal membrane removal, membrane peeling, endolaser, silicone IOL injection, belt buckling, transcleral cryo, octofluoro propane gas filling. Group II–lensectomy combined with other procedures, Group III–intravitreal injection of Avastin, Group IV–silicone oil removal, Group V–glaucoma surgical procedures [Table 2].

**Table 1:** Number of postoperative infections categorized according to the time duration: Data from 2000 to 2007

Category	Type of surgery		
	No. of infective endophthalmitis		
	Cataract	PK	Other intraocular surgeries
Acute onset	67 (0.05)	8 (0.4)	15 (0.015)
Late onset (after 6 weeks)	3 (0.002)	2 (0.1)	3 (0.03)
After 1 year	-	-	-
Total: 98 cases	70 (0.053)	10 (0.01)	18 (0.018)

Figures in parentheses are in percentage

**Table 2:** Postoperative infectious endophthalmitis resulted following surgeries performed other than cataract and penetrating keratoplasty and the microbial pathogen isolated from the corresponding diagnostic specimen

Category	Surgery	Microorganism isolated
Acute onset Group 1 Vitrectomy # of patients 9 (043%)	Patient 1	*S. epidermidis*
	Patient 2	*Alkaligenes faecalis*
	Patient 3	*S. aureus*
	Patient 4	*P. stutzeri*
	Patient 5	*P. stutzeri*
	Patient 6	*Staph. aureus*
	Patient 7	*Acinetobacter spp*
	Patient 8	*S. epidermidis*
Group II Lens aspiration # of patients 2 (0. 029%)	Patient 1	*P. stutzeri*
	Patient 2	*P. stutzeri*
Group III # of patients 1(0.14%)	Patient 1	*Staph. aureus*
Group IV Silicone oil removal # of patients 1 (0.018%)	Patient 1	*S. epidermidis*
Group V Surgery for glaucoma # of patients 2 (0.026%)	Patient 1	*S. aureus*
	Patient 2	*C. species*
Late onset (after 6 weeks) Group 1 Vitrectomy # of patients 3 (0.014%)	Patient 1	*S. epidermidis*
	Patient 2	*A. flavus*
	Patient 3	*Klebsiella spp*
Total: 18

The incidence of postoperative infectious endophthalmitis following lensectomy was 0.029% (2/, 6669), vitrectomy acute onset–0.043% (9/ 20835), chronic onset 0.014% (3/ 20835), intravitreal Avastin injection–0.14% (1/ 717) and silicone oil removal–0.018% (1/ 5356).

**Figure 1:**
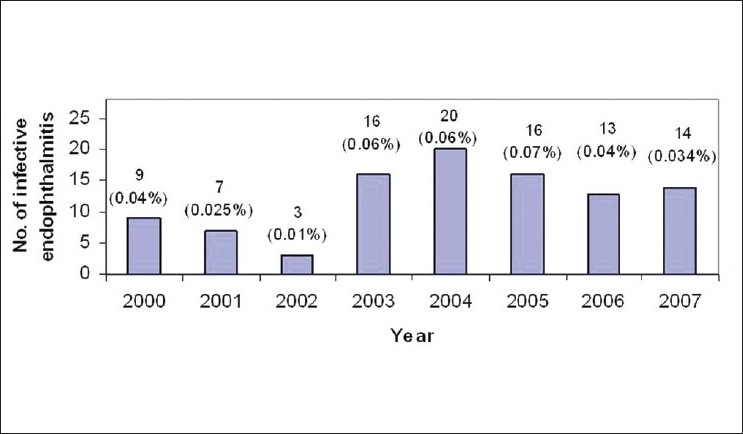
Number of postoperative infectious endophthalmitis and the percentages of the same for each year. The percentages varied from 0.01 to 0.07, with an average of 0.042%

The number of postoperative infectious endophthalmitis and the percentages of the same for each year are shown in Fig. 1. Analysis of year-wise distribution of 98 postoperative infectious endophthalmitis showed a maximum of 0.07% in 2005 to 0.01% in 2002, with an average infection rate of 0.042%.

**Figure 2:**
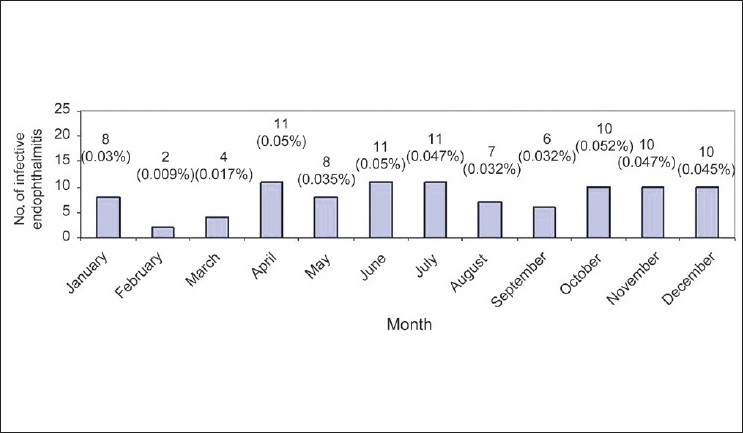
Ninety-eight postoperative endophthalmitis during the years 2000 and 2007 distributed according to the month when the surgery was performed. The average percentage for each of the months is shown in parenthesis. The lowest rate of postoperative infectious endophthalmitis was during February and during all other months, the rate varied from 0.017 to 0.05%, indicating only sporadic occurrence without any outbreak

Distribution of 98 postoperative infectious endophthalmitis during the years 2000 and 2007 according to the months when the surgeries were performed along with the average percentages of its occurrence for each of the months in parenthesis are shown in Fig. 2. The lowest infection rate was observed during February, with rates varying from 0.017 to 0.05% during all other months, indicating only a sporadic occurrence without any outbreaks during these 8 years.

The list of microorganisms isolated from the 98 patients is shown in Table 3. Bacteria were the most common cause of postoperative infectious endophthalmitis, being 87 (89.7%) Gram-positive (44) and Gram-negative (43) bacteria equally distributed among them. The predominant Gram-positive bacteria were *Staphylococcus* spp (30 of 44 patients). Among Gram-negative bacteria, *Pseudomonas* spp. (19) formed the major bulk, with several others distributed in smaller numbers. Polymicrobial infection was noted in four patients, fungi isolated in seven patients and *Aspergillus flavus* in five others.

**Table 3:** List of microorganisms isolated from the intra-ocular specimens collected from the 98 patients with postoperative infectious endophthalmitis during the 8-year period (2000–2007)

Groups of microorganisms		Names of the microorganisms	Cataract surgery	PK	Others
Bacteria (87)	Gram-positive (44)	*S. epidermidis* (17)	13		4
		*S. aureus* (13)	8	1	4
		*Micrococcus* spp. (1)	1	1	1
		*Streptococcus* spp.(3)	2	2	
		*E. faecalis* (3)	1		
		*Corynebacterium* spp. (3)	2		
		*B. cereus* (2)	2		
		*Nocardia* spp. (2)	2		
	Gram-negative (43)	*P. aeruginosa* (7)	4	2	1
		*P. stutzeri* (12)	7	1	4
		*A. faecalis* (9)	7	1	1
		*Klebsiella* spp.(4)	2	1	1
		*Acinetobacter* spp (6)	5		1
		*Citrobacter* spp. (3)	3		
		*Enterobacter* spp.(1)	1		
		*Flavobacterium* spp. (1)	1		
Fungus (7)		*Aspergillus flavus* (5)	3	1	1
		*Candida albicans* (1)	1		
		*Phialomonium dimorphosporum* (1)	1		
Polymicrobial (bacterial and fungal) (4)		*Micococcus* spp. and *Acinetobacter* spp. (1)	1		
		*Enterobacter* spp. and *Trichosporon* spp. (1)	1		
		*Bacillus* spp. and *E. faecalis* (1)	1		
		*S. epidermidis* and *Acinetobacter* spp. (1)	1		
			70 (0.05%)	10	18

Infective agents isolated from 10 patients who had postoperative infections following PK included *Enterococcus faecalis*-3, *Pseudomonas aeruginosa*-2, Methicillin-resistant *Staphylococcus aureus* (MRSA)-1, *Alkaligenes faecalis*-1, *Aspergillus flavus*-1, *Klebsiella pneumoniae*-1 and *Pseudomonas stutzeri*-1. Among these 10, the source of infection could be established as donor corneal scleral rims in six (60%) patients since they showed growth of E. *faecalis*-2, *P. aeruginosa*-2, MRSA-1 and *K. pneumoniae*-1, with the phenotypic characteristics identical with the corresponding bacterium isolated from the diagnostic intra-ocular specimens collected from the respective patients. In another patient, *Corynebacterium* spp. was isolated from the aqueous humor and investigation on the cytotoxicity test on the viscoelastic solution of the batch and the lot no. used for the patient during surgery was positive. We believe that intra-ocular inflammatory fibrin reaction produced by the toxicity of the viscoelastic solution may have allowed the bacterium to be caught up in the fibrin, resulting in its multiplication with development of endophthalmitis. During this period, noninfectious inflammation due to viscoelastic toxicity was encountered in one patient.

## Discussion

The present report is a prospective, 8-year study, well-documented in a specifically formatted form in a high-volume ophthalmic surgical center related to the incidence and microbiology of postoperative infectious endophthalmitis. The study showed the lowest documented incidence (0.042%) of postoperative infectious endophthalmitis compared with similar reports published in literature. Other reports comparable to our study are a review by Katten *et al*.,[15] who reported 0.079% of hospital-linked postoperative endophthalmitis and another by Aaberg *et al*., who reported the overall incidence of postoperative endophthalmitis at 0.093%.[16]

With specific reference to postcataract infectious endophthalmitis, Aaberg *et al*. reported the incidence of 0.082% over a 10-year period.[16] In a meta-analysis, an overall postoperative infection rate of 0.27% following cataract extraction was reported.[17]

In a multicentric study conducted on the prophylactic effect of intracameral cefuroxime injection (group I)/perioperative levofloxacin in (group II) eye drops, the incidence of endophthalmitis was higher among group I (0.4%) compared with group II (2.0%).[18] Mortel *et al*. have reported the incidence of postoperative endophthalmitis to have ranged between 0.65 per 1000 operations and 16.4 per 1000 operations over 19 years.[19] In another review, an overall incidence of infection rate of 0.128% was reported.[20] Patwardhan *et al*. have reported an incidence rate of 0.36% post-PE surgeries.[21] Compared with all these reports on the incidence rate of postoperative endophthalmitis following cataract surgery, our 8-year study was among the lowest, at 0.055%. Only other report that showed a lower rate of incidence (0.03%) was from south India in a 2-year study of postcataract infectious endophthalmitis.[4]

With reference to post PK infectious endophthalmitis, our study indicated a higher rate of incidence at 0.5% compared with those reported in literature. In a major review, an overall estimate of 0.382% post-PK endophthalmitis was reported.[22] An incidence of 0.194% combined post-PK and postcataract endophthalmitis was reported by Aaberg *et al*.[16] Kattan *et al*. reported an infection rate of 0.11% following PK.[15] A higher incidence of post-PK infectious endophthalmitis in the present study is probably due to the inadequate clearance of the bacterial load in the donor cornea during preservation.

Kattan *et al*.[15] and Aaberg *et al*.[16] reported an incidence of 0.051 and 0.046% culture-proven endophthalmitis following post pars plana vitrectomy, respectively. Our study has recorded 0.026% of postsurgical infectious endophthalmitis in similar intra-ocular surgeries.

Analysis of microbiological investigations to establish the source of infection in all the 98 postoperative endophthalmitis could yield useful results in post-PK endophthalmitis and in one PE surgery. The source of *Pseudomonas stutzeri* in a post-PE patient with infectious endophthalmitis was identified as phacoemulsification probe because genotypically similar *Pseudomonas stutzeri* was isolated from both the vitreous aspirate and the washings of the phaco probe used for the patient. We have reported this earlier.[14] In 60% of the post-PK surgeries, the donor buttons were considered the likely source of infection because of growth of phenotypically similar organisms from the donor corneal rims of the donor eyeball used and the intra-ocular specimens of the patients, indicating that the microbial load of the corneal button was not reduced during the preservation.

We believe that the overall lower rate of incidence of postoperative infectious endophthalmitis observed at our hospital is due to the periodic monitoring of the OTs for microbial load maintenance of proper functioning of autoclaves and other sterilizers and several other procedures described above in the OT. The microbial load in the 22 OTs monitored during the most highly activity period every 15 days as planned at the beginning of each year by settle plate method and slit air sampling method was within ≤ 180 colony forming unit (CFU)/mm^2^ /min for the settle plate method and count of ≤ 35 CFU/m^2^ /min for the slit air sampling method permissible limits). The policy of the use of prophylactic sulphacetamide eye drops for 3 days before the planned surgery probably had a role in the reduction in the microbial load of conjunctiva. We believe that the prophylactic injection of ampicillin–sulbactum before surgery may have had bactericidal activity on bacteria entering into the anterior chamber. Because sulphacetamide eye drops are not commonly used in community practice and with evidence of least drug resistance of the bacterial flora of conjunctiva to this drug, we believe presurgical bacterial load in the conjunctiva is reduced to a large extent. Because compliance of the use of prophylactic eye drops by the patients has often been questionable, we felt that the prophylaxis was ensured with injection of ampicillin-sulbactum before surgery. Ciulla *et al*. have reported the prophylactic effect of a pre-operative povidone-iodine preparation in their review.[23]

With regard to microbiological investigations in this study, bacteria were the most common cause, forming 89.7% of the isolates, with the rate of isolation of Gram-positive organisms and Gram-negative bacteria being equal. Reports from the western literature indicated that Gram-positive bacteria, particularly *Staphylococcus* spp., were the most common cause of postoperative endophthalmitis[24,15] In India, Kunimoto *et al*. have reported a high prevalence of Gram-negative species and fungi apart from Gram-positive bacteria.[25] In an earlier study, we reported an isolation rate of 41.7% of Gram-negative and 37.6% of Gram-positive bacteria and 21.8% of fungi.[26] *Nocardia* spp. was reported as the predominant bacteria isolated from patients with postcataract endophthalmitis from south India.[4] These reports suggest that empiric therapy should include coverage for both Gram-positive, including Actinomycetes, and Gram-negative pathogens and for fungal pathogens in appropriate settings. Any preventive strategy should consider these facts in our setting in India.

The major drawback of the study was that the source of the infectious agent of postoperative endophthalmitis could be proven in only seven among the 98 patients and in others, the probability of the source of infection is presumed as the conjunctival sac of patients or environmental since no specific investigation was possible to prove their sources.

In conclusion, the average of postoperative infection rates of the periods 2000-2007 in our center was lowest compared with others reported and we could establish the source of infection in seven cases. As donor corneal rims and phaco probes are a potential source of postoperative endophthalmitis, donor corneal rims and phaco probes should be cultured in case of post-operative endophthalmitis following PK and phaco emulsification surgeries to detect the possible source of infection. The empiric therapy for postoperative endophthalmitis should cover both Gram-positive and Gram-negative organisms as they are grown in equal proportions.
